# Body Mass Index and Physical Fitness among Chinese Adolescents Aged 15–18: A Cross-Sectional Study of Gender Differences

**DOI:** 10.3390/children10071204

**Published:** 2023-07-12

**Authors:** Guoqing Liu, Rongkai Hao, Xiaotian Li, Yucheng Gao, Wenjie Li, Meijie Zhang

**Affiliations:** 1 Institute of Physical Education and Training, Capital University of Physical Education and Sports, Beijing 100191, China; liuguoqing2022@cupes.edu.cn (G.L.);; 2 School of Recreation and Community Sport, Capital University of Physical Education and Sports, Beijing 100191, China

**Keywords:** adolescents, body mass index, health, obesity, physical fitness

## Abstract

Objective: The aim of this study is to examine the relationship between varying levels of obesity and physical fitness test scores among Chinese adolescents, while also investigating gender differences in physical fitness and overall health. Data on height, weight, 50 m sprints, 1000/800 m runs, standing long jumps, sit and reach exercises, pull-ups/bent-leg sit-ups, and additional variables were collected from the DYH health database. Physical fitness indicators were evaluated based on the revised 2014 National Physical Fitness Standards for Students, and scores were assigned accordingly. An ordinal logistic regression (ologit) model was employed to analyze the relationship between body mass index (BMI) and physical fitness. Results: (1) Normal-weight boys achieved significantly higher physical fitness test scores than boys in other weight categories. (2) Underweight and normal-weight girls obtained significantly higher physical fitness test scores compared to overweight and obese girls, with underweight girls scoring slightly higher than those with a normal weight. Conclusions: (1) There is a significant non-linear relationship between BMI levels and physical fitness test scores. (2) Gender differences were observed in physical fitness and overall health outcome. (3) The findings indicate an inverted U-shaped association between BMI and physical fitness among boys, while girls displayed an inverse relationship. This could be attributed to the cultural association between thinness and beauty among girls in China.

## 1. Introduction

The World Health Organization (WHO) reported in 2021 that more than 340 million children and adolescents between the ages of 5 and 19 are currently overweight or obese, compared with less than 1% of children and adolescents between the ages of 5 and 19 who were obese in 1975 [1]. For children and adolescents, an average of 60 min per day of moderate-to-vigorous intensity aerobic physical activity across the week provides health benefits [2]. As the economy and technology continue to develop, Chinese adolescents are faced with the challenges of increasing obesity and physical inactivity [3,4,5] and, as a result, the physical health of Chinese students is in decline [6], and the malnutrition of adolescents is more serious [7]. Fitness testing serves as a fundamental means to assess the physical fitness of adolescents, and plays a positive role in promoting their overall health. Schools, guided by the National Student Physical Fitness Standards (CNSPFS) [8], conduct comprehensive fitness tests to gain a holistic understanding of students’ physical well-being. These tests encompass various aspects, including aerobic endurance, muscular strength, flexibility, and other indicators, with the aim of evaluating students’ overall physical fitness levels. Through these tests, schools can obtain quantitative data on students’ physical fitness, enabling them to identify potential health issues and intervene promptly.

Given that more adolescents are experiencing problems with subhealth [2], by exploring the relationship between body mass index (BMI) and physical fitness in adolescents, we can enhance our understanding of youth health issues. Obesity and declining physical fitness have become significant health concerns for adolescents in modern society. This study aims to provide empirical data on the correlation between BMI and physical fitness, thereby raising awareness of youth health issues in society. Specifically, this study focuses on the relationship between adolescent physical health and BMI indicators, in the context of promoting a healthy China. However, there are several aspects that differentiate this study from previous research.

Firstly, previous studies mostly examined the changes in BMI and physical fitness using means and t-tests, which do not directly reflect the relationship between BMI and physical health. In this study, the original data from physical fitness tests were transformed into corresponding scores, according to the established standards. These scores reflect the students’ health levels, where higher scores indicate better health. Thus, the use of physical fitness scores provides a more intuitive reflection of the relationship between BMI and physical health. Secondly, this study specifically focuses on Chinese adolescents aged 15–18, addressing a research gap within this specific population. While there have been studies on the relationship between BMI and physical fitness, most of them were conducted in other countries or regions. By focusing on Chinese adolescents, this study fills the research gap in examining the relationship within this specific population, and provides targeted data and insights into youth health issues in the region. This has significant implications for the development of physical fitness in this population. Thirdly, gender differences play a crucial role in the relationship between BMI and physical fitness, which has often been overlooked in previous research. By paying attention to gender differences, this study aims to uncover the disparities between the genders in terms of BMI and physical fitness. It provides a scientific basis for gender-specific physical education and training. One of the innovations of this study is to address the research gap in gender differences within the study of BMI and physical fitness, offering new perspectives and theoretical foundations for the academic and practical fields. Fourthly, unlike previous studies that focused on a single physical fitness indicator, this study comprehensively considers multiple physical fitness indicators, including the 50 m sprint, 1000/800 m run, sit and reach, pull-up, and bent-leg sit-up performances. This comprehensive approach allows for a more comprehensive evaluation of the physical fitness levels of adolescents, and reveals the relationship between BMI and multiple physical fitness indicators. By considering multiple physical fitness indicators, this study provides more accurate and comprehensive data to better understand the relationship between BMI and youth physical fitness. Furthermore, previous studies did not analyze male and female students separately. Therefore, this study conducts separate analyses for male and female students, examining their performance in different physical fitness indicators and comparing the differences among different weight categories. This gender-specific analysis helps with understanding the gender differences in physical fitness performance, and serves as a reference for designing gender-specific fitness programs and interventions.

Finally, it investigates the relationship between BMI categories (underweight, normal weight, overweight, and obese) and physical fitness test performances among mainland Chinese adolescents. It attempts to reveal the relationship between body mass index and physical health by employing the ologit (ordinal logit) correlation test, while also taking into account robustness checks to avoid statistical errors.

## 2. Materials and Methods

### 2.1. Fitness Test

#### 2.1.1. 50 m Sprint

Participants were grouped into groups of two or more for testing. The sprint started from a standing position, covering a distance of 50 m. Upon hearing the command “go”, the participants began to sprint. The timekeeper started the stopwatch upon the command while simultaneously waving the flag. The time was stopped when the participants’ torsos crossed the vertical plane of the finish line. The test results were recorded in seconds, with a precision of one decimal place. The second decimal place was rounded up using the non-zero rounding rule. For example, 10.11 s would be recorded as 10.2 s.

#### 2.1.2. 1000 m/800 m Run

Participants were grouped for testing, starting from a standing position. Males ran a distance of 1000 m, while females ran 800 m. The run began upon hearing the command “go”. The timekeeper started the stopwatch upon seeing the flag move and stopped it when the participants’ torsos crossed the vertical plane of the finish line. The test results were recorded in minutes and seconds, without decimals.

#### 2.1.3. Standing Long Jump

Participants stood with their feet naturally apart, behind the starting line, ensuring that the toes did not touch the line. They jumped forward with both feet simultaneously, without any additional steps or consecutive jumps. Each person made two jumps, and the best result was recorded.

#### 2.1.4. Sit and Reach

Participants sat on the testing device with their legs straight, bare feet pressed against the measurement scale, and their upper bodies leaning forward. With arms extended straight ahead, they gradually and steadily pushed the sliding board forward using their fingertips until they could not reach further.

#### 2.1.5. Pull-Ups

Participants grabbed the bar with an overhand grip and hands shoulder-width apart, hanging with straight arms. After achieving a stationary position, they exerted force simultaneously with both arms to pull themselves up until their chin surpassed the upper edge of the bar. The number of pull-ups was recorded.

### 2.2. Data

The data were obtained from the Database of Youth Health (DYH) [9]. The DYH database utilized on-site testing, survey questionnaires, and data collection methods to obtain data on adolescent health resources, including multiple waves of surveys conducted during the academic years of 2015/2016, 2016/2017, 2017/2018, and 2020/2021. These surveys aimed to investigate the current status of health and health-related behaviors among Chinese middle and high school students. This dataset represents the first openly shared data collection on the health and health-related behaviors of Chinese adolescents. It would be valuable and beneficial for policymakers, educational institutions, and other stakeholders to develop or adjust existing strategies to improve the well-being of Chinese youth [9]. The data includes physical fitness test data and body composition data of adolescents from 17 cities in Shandong Province for the years 2015–2020 [10]. Abnormal data were eliminated according to the following principles: (1) abnormal data that produced significant deviations from the actual situation due to the measurement and entry process; and (2) data missing from the database. Valid data from a total of 12,576 participants were collected, numbering 6099 male students (48.5%) and 6477 female students (51.5%). The sample age ranged from 15 to 18 years old, covering areas with different levels of economic development in Shandong, which is highly representative. The surveyed students all gave informed consent and met ethical requirements. Student test data are shown in Table 1 and Table 2.

### 2.3. Research Methods

Physical fitness tests are assessed using the National Student Physical Fitness Standards [8] (revised in 2014) (CNSPFS), which measures different components of physical fitness and can be divided into three categories according to the Standards’ test items: (1) body shape (body shape can be classified as 1—underweight, 2—normal weight, 3—overweight, and 4—obese, based on body mass index); and (2) physical function (physical fitness (50 m sprint, 1000/800 m run, standing long jump, sit and reach, pull-up/bent-leg sit-up). All indicators are measured and monitored in accordance with the National Student Physical Health Implementation Plan and related rules, and students’ height, weight, and physical fitness are measured and monitored.

The physical fitness test was selected from physical fitness test items, specifically the 50 m sprint, 1000/800 m run, standing long jump, sit and reach, and pull-up/bent-leg sit-up; 5 items required in the Standards to test for students’ aerobic endurance, upper limb muscle strength, and muscle for boys, respectively. The test was conducted by the physical education teachers in strict accordance with the physical fitness standards.

Body mass index, known as BMI (weight kg/height m^2^), is currently an internationally accepted method of determining the degree of obesity in the human body [11]. BMI is widely used to assess an individual’s weight status, and serves as a tool to indicate whether they are underweight, normal weight, overweight, or obese.

### 2.4. Statistical Analyses

The data from the database of adolescents in Shandong Province were statistically and analytically analyzed using stata17 software, and the raw data of the physical fitness test were given corresponding scores (0/10/20/30/40/50/60/70/80/90/100) according to the CNSPFS criteria [8]. Based on the student body mass index (BMI) values, and in strict accordance with the requirements of the criteria [8], students were classified into four levels of body shape: (1) underweight, (2) normal weight, (3) overweight, and (4) obese (Table 3).

According to the nature of the variables, the correlation between BMI and physical fitness test scores of students with different levels of obesity was analyzed by gender, using ordered logistic regression models (referred to as ologit). In the senior year of study in China, where there is a heavy academic load and possible abnormalities in the fitness test, the sample size was reduced for robustness testing. The results showed that they passed the robustness test (Table 4).

## 3. Results

### 3.1. Gender Differences in the Relationship between BMI and 50 m Sprint Performance

**Figure 1:**
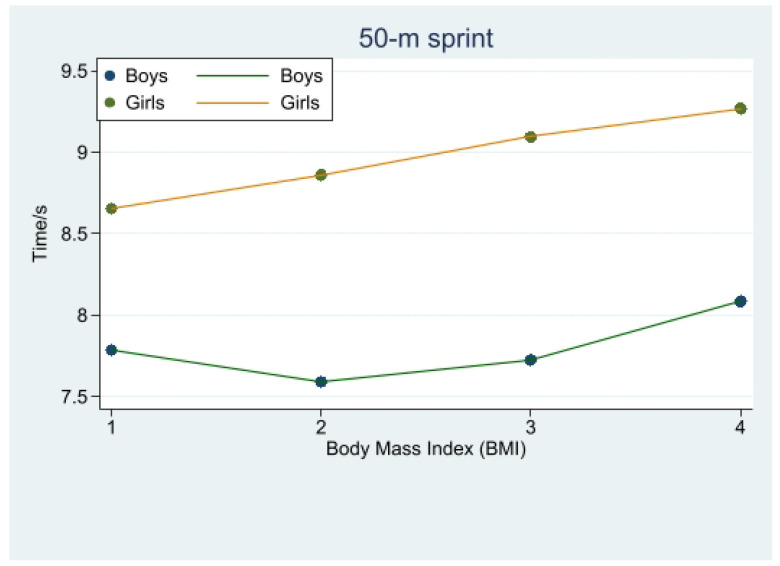
Change in 50 m sprint time with body mass index.

In the 50 m sprint performance of male students, obese and overweight, underweight, and normal weight had significant differences (*p* < 0.001), and normal weight and other weights showed significant differences (*p* < 0.001), specifically: normal-weight male students performed the best in 50 m sprint performance, followed by overweight students, and underweight students performed significantly better than obese students. (Figure 1, Table 3).

In terms of girls’ 50 m sprint performance: girls with the obese body type had a significant difference (*p* < 0.001) with underweight and normal-weight girls, while girls with overweight and obese body type also had a difference (*p* < 0.05) in their 50 m sprint performance, but performed worse compared to normal-weight and underweight girls. This phenomenon indicates that normal-weight as well as underweight girls performed better in the 50 m sprint, and the data showed a significant difference between underweight and normal-weight girls in the 50 m sprint scores, with underweight girls performing the best. The girls with overweight and obese forms also had differences in 50 m sprint scores (*p* < 0.05), but their scores were worse compared to those of underweight and normal-weight girls. (Figure 1, Table 3).

### 3.2. Gender Differences in the Relationship between BMI and 1000/800 m Run Performance

In the 1000 m run performance of boys, there was no significant difference between normal-weight and underweight students, but the overall mean value of the performance of normal-weight boys was better than that of underweight ones. The difference between the overweight and the obese boys was not significant, while there was a significant difference between the obese and the underweight boys (*p* < 0.01), and there was a significant difference between the normal-weight boys and the obese boys in the 1000 m run (*p* < 0.001). This difference indicates that boys with a body mass index in the normal range had the best performance in the 1000 m run, followed by underweight students, and those who were overweight performed better than those who were obese, but the difference was not significant.

**Figure 2:**
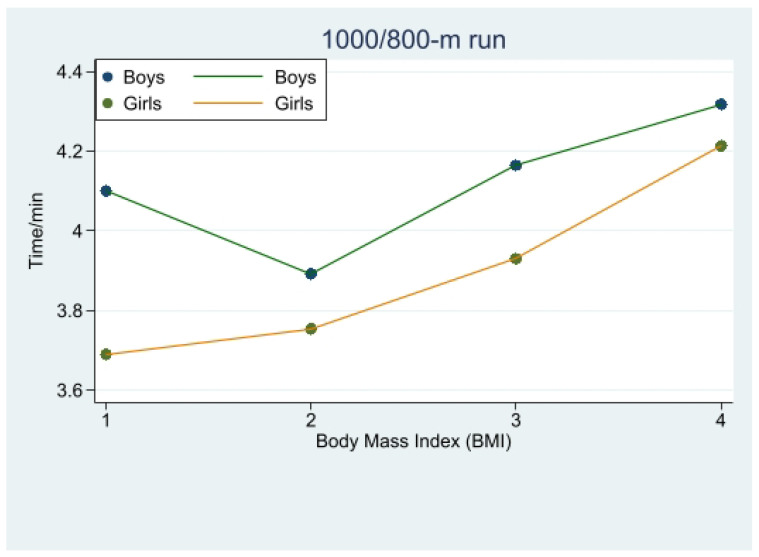
Change in 1000/800 m run time with body mass index (BMI).

In the 800 m run of girls, there is a significant difference between obese and underweight girls and normal-weight girls (*p* < 0.001), and between obese and overweight girls (*p* < 0.05). This further indicates that the correlation between body mass index and 800 m run was highly significant among high school girls; specifically, underweight and normal-weight girls performed better in the 800 m run. Also, underweight students performed slightly better than normal-weight girls in endurance qualities (Figure 2, Table 3).

### 3.3. Gender Differences in the Relationship between BMI and Standing Long Jump Performance

In the standing long jump, there was, firstly, a highly significant difference between obese body type boys and overweight, underweight, and normal-weight boys (*p* < 0.001). Secondly, there was a significant difference (*p* < 0.001) between all the normal-weight students and the other weight students. It is evident from the data that the normal-weight boys had the best performance in the standing long jump, followed by the underweight students, closely followed by the overweight body type students, and the obese students had the worst performance.

**Figure 3:**
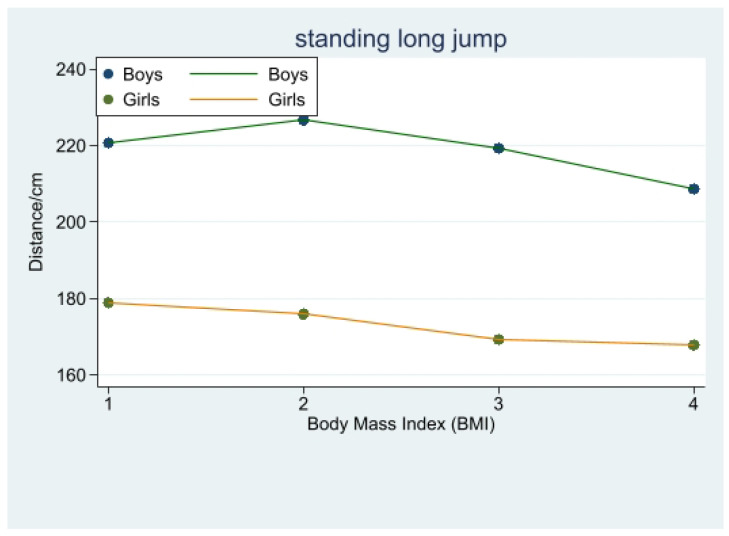
Change in standing long jump distance with body mass index (BMI).

In terms of female standing long jump performance, there was a significant difference between obese girls and underweight and normal-weight girls in standing long jump performance (*p* < 0.001), no significant difference between obese and overweight girls (*p* > 0.05), and underweight girls performed significantly better than normal-weight ones (Figure 3, Table 3).

### 3.4. Gender Differences in the Relationship between BMI and Sit and Reach Scores

In terms of boys’ sit and reach performance, there was a significant difference between obese boys and overweight and normal-weight boys (*p* < 0.001), but the difference between obese and underweight students in sit and reach performance was not significant (*p* > 0.05). As can be seen from the graph, normal-weight students had the best performance, followed by overweight students, closely followed by underweight and obese students.

**Figure 4:**
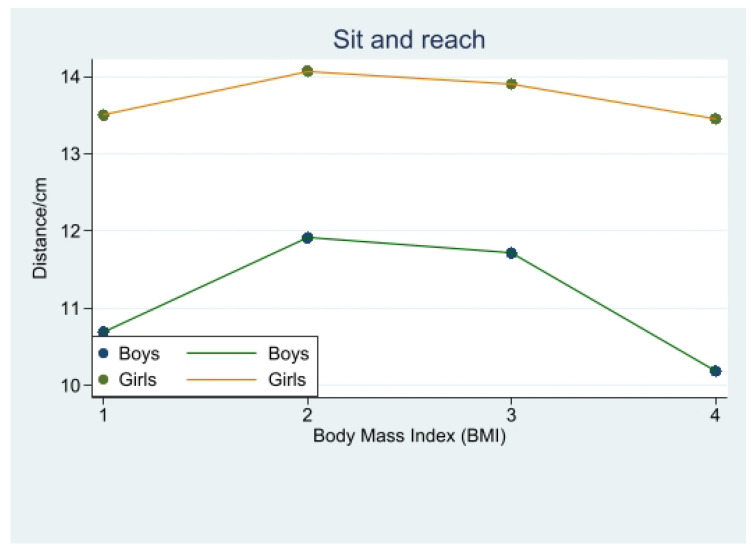
Change in sit and reach distance with body mass index (BMI).

There was no significant difference between obese girls and overweight and underweight girls in the sit and reach performance (*p* > 0.05), but there was a significant difference when compared with normal-weight students (*p* < 0.05), which indicated that normal-weight students performed best in the sit and reach (Figure 4) (Table 3).

### 3.5. Gender Differences in the Relationship between BMI and Pull-Up and Bent-Leg Sit-Up Performance

There was a significant difference in pull-up scores between obese male students and overweight, underweight, and normal-weight students (*p* < 0.001), with no statistically significant difference in scores between normal-weight and underweight boys. Specifically, underweight students had the best scores, followed by normal-weight students, closely followed by overweight body types, and obese students had the worst results.

**Figure 5:**
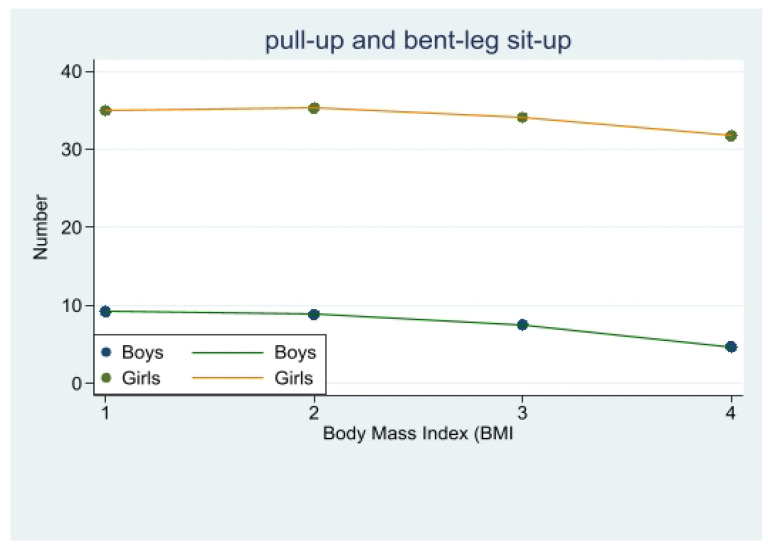
Changes in the number of pull-ups and bent-leg sit-ups with body mass index (BMI).

There was no significant difference in bent-leg sit-up between overweight and obese girls (*p* > 0.05), a significant difference between obese girls and underweight and normal-weight girls (*p* < 0.001), a significant difference between normal-weight girls and overweight girls (*p* < 0.05), but no significant difference with underweight girls. Specifically, underweight girls had the best performance, followed by normal-weight, then overweight, and obese girls had the worst performance. (Figure 5, Table 3).

## 4. Discussion

Physical fitness test scores play a vital role in assessing students’ overall health, as higher scores are typically associated with better physical well-being. However, it is important to consider potential gender differences in physiological and psychological mechanisms when analyzing these scores. Therefore, this study aims to separately analyze the relationship between body mass index (BMI) and physical fitness test scores for male and female students. By doing so, we can examine the influence of gender on the observed outcomes. Examining this relationship will provide insights into how different BMI levels and gender influence the physical fitness test scores of adolescents. Additionally, it will enable us to predict test performance based on individuals’ BMI and gender, and offer recommendations for enhancing both test scores and overall physical health using gender-specific BMI classifications.

### 4.1. Exploring the Relationship between Body Mass Index and 50 m Sprint Performance in Different Genders

In terms of the 50 m sprint performance, normal-weight boys were found to be faster than both underweight and overweight boys. This observation could be attributed to the fact that being either thin or obese impairs students’ speed performance [12]. Overweight students performed better than underweight students, which may be due to a higher BMI reflecting an increase in non-fat mass rather than fat mass [13,14]. For instance, athletes might be categorized as overweight based on BMI, even though they do not have excessive fat. Kryst et al. [15]. suggested that overweight individuals typically possess more robust bones and greater muscle mass than underweight individuals, which could account for the superior 50 m sprint scores among overweight students compared to underweight students. However, normal-weight participants demonstrated better results in the physical fitness test, due to a higher proportion of bone and muscle mass relative to their overall body weight [15].

In adolescent girls, both underweight and normal-weight individuals achieved better results in the 50 m sprint performance compared to overweight and obese girls [16]; the girls’ 50 m sprint performance exhibited a negative correlation with BMI, with an increase in BMI leading to a decrease in sprint performance. The best 50 m sprint performance for males was observed in the normal body mass group, while for females, it was the underweight group. This difference may be related to the generally higher fat content in females compared to males, suggesting a strong correlation between 50 m sprint performance and students’ body mass index. In summary, male students with a body mass index within the standard range tend to have significantly better 50 m sprint performances compared to those with other BMI values. On the other hand, female students with an underweight body mass index demonstrate significantly better 50 m sprint performances than their counterparts in other BMI categories. This highlights the importance of maintaining a healthy body mass index for optimal physical fitness and performance among adolescents.

### 4.2. Exploring the Relationship between Body Mass Index and 1000/800 m Run Performance in Different Genders

In the present study, we found that the 1000 m run time was significantly longer for both overweight and obese boys, compared to normal-weight and underweight boys. No significant difference was observed between underweight and normal-weight boys, which is in line with the study by Sekulice et al. [17]. Sziva explained that an increase in body fat content can lead to a decrease in cardiorespiratory endurance [18]. In boys, both underweight and overweight adolescents demonstrate inferior endurance qualities compared to their normal-weight counterparts [19,20]. Some researchers have noted [21] a negative correlation between BMI and 1000 m run performance, with better performance associated with a lower rate of overweight and obesity. This could be attributed to adolescents with good endurance qualities engaging in regular physical activities, thus reducing the likelihood of obesity.

Interestingly, with girls, our study revealed that underweight individuals performed better than normal-weight girls [22,23]. The time spent by underweight students was significantly less than the time spent by overweight and obese students. Mehta et al. [20] reported that overweight and obese girls exhibit worse endurance qualities than normal-weight females. Gentier et al. [24] suggested that obesity impairs motor performance by compromising the precise control required for exercise. Obesity-related damage to nerves in cortical areas associated with motor function could contribute to poor 800 m run performances among obese individuals [25].

### 4.3. Exploring the Relationship between Body Mass Index and Standing Long Jump Performance in Different Genders

The standing long jump is an important indicator of lower limb explosive power, and our study found that normal-weight boys performed best in this regard, which aligns with research by Mendoza et al. [26]. Sung et al. claimed that overweight children have superior strength performance [27]. However, overweight adolescents exhibit weaker relative lower limb explosive power due to their increased body weight [28], indicating that overweight individuals performed worse than normal-weight and underweight individuals in standing long jump performance. In the case of boys, underweight students in this study had poorer performance, and Bovet et al. suggested [23] underweight adolescents possess weaker muscle strength, leading to lower standing long jump performance than normal-weight peers. Our study also revealed that overweight boys demonstrated significantly better standing long jump performance than obese boys. Other studies have indicated [29,30] that, within a certain range, increased weight among adolescent boys may be due to an increase in muscle mass, contributing to improved standing long jump performance among overweight students. This phenomenon has been identified by other researchers as well [23]. For boys, weight gain at lower BMI levels is mainly due to increased body muscle mass, while at higher BMI levels, it primarily reflects increased body fat. Therefore, both underweight and obese individuals demonstrate negative impacts in standing long jump performance.

Regarding girls, our study aligns with findings by Verney et al. [16,31]: underweight individuals exhibit superior performance compared to normal-weight, overweight, and obese individuals. However, the difference between normal-weight and underweight girls in this study was not statistically significant. Verney et al. also concluded [28,32] that obese adolescents exhibit better performance in static strength exercises, but worse performance in dynamic or explosive power tests. Artero et al. [28] further noted that BMI demonstrates a negative correlation with standing long jump performance, with increasing BMI having a greater negative impact on lower limb explosive power. Therefore, when girls are overweight, their relative strength decreases, leading to a poorer standing long jump performance.

### 4.4. Exploring the Relationship between Body Mass Index and Sit and Reach Performance in Different Genders

This study revealed that normal-weight students performed best in the sit and reach test. For boys, Chen et al. [33] found that overweight students had better scores in the sit and reach test than underweight and obese students, consistent with our results. Additionally, underweight adolescents had poorer scores in the sit and reach test than normal-weight adolescents [34].

Among female students, normal-weight students outperformed overweight students in the sit and reach test, while no significant differences were observed among other weight categories in terms of flexibility. The body mass index had a significant impact on students’ sit and reach scores, as weight gain and abdominal fat accumulation can limit lumbar movement and affect bending forward movements, leading to poorer sit and reach test scores and flexibility among obese individuals [34].

### 4.5. Exploring the Relationship between Body Mass Index and Pull-Up and Bent-Leg Sit-Up Performance in Different Genders

Pull-ups are an indicator of the upper limb muscle groups, shoulder girdle muscle groups, and power strength endurance [35]. In this study, we found that pull-up performance of normal-weight and underweight boys was significantly better than overweight and obese body type boys, and pull-up performance of overweight body type boys was significantly better than obese body type boys, while the difference between normal-weight and underweight boys was not statistically significant, which is consistent with the study by Mak et al. [36,37]. Pull-ups are a strength exercise method to overcome one’s own body weight, so weight is an important factor in performance, and being overweight and obese can seriously affect pull-up performance.

The study in this paper showed that underweight and normal-weight students performed better, and overweight and obese body type students performed worse [36]. Scholars believe that the bent-leg sit-up performance of adolescents is influenced by a combination of body weight and muscle [38,39]. In obese individuals with high abdominal fat content, the work performed by the body to overcome gravity increases, and excess fat also limits the ability of muscles to sustain oxygen supply [40]. Therefore, fat content has a significant effect on sit-up performance, which is what causes the poor performance of overweight and obese individuals in this paper.

In summary, there is a significant correlation between body mass index (BMI) and various physical qualities, such as aerobic capacity, flexibility, muscular explosive power, speed qualities, and muscular endurance among adolescents. Overall, the following observations can be made: Firstly, both obese males and females exhibit lower levels of physical fitness across all aspects. Secondly, among boys, individuals with higher body weight may possess greater muscular strength and demonstrate better performance in measures of muscular explosive power and speed compared to obese individuals. However, the differences between overweight girls and obese individuals in various aspects are not significant. Thirdly, underweight boys show good levels in speed, muscular explosive power, and muscular endurance qualities, but their performance in aerobic capacity and flexibility is unsatisfactory. Underweight girls, on the other hand, demonstrate high levels in speed, aerobic capacity, muscular explosive power, and muscular endurance, while their performance in flexibility is average. Fourthly, boys with a normal weight exhibit high levels of performance across all aspects. Normal-weight girls, however, do not perform as well as underweight girls in measures reflecting lower limb muscle strength, but excel in other aspects. Notably, our study reveals heterogeneity between male and female students, with normal-weight boys achieving the highest scores, and underweight girls demonstrating the best performance. This finding is novel, and may be attributed to the cultural association between thinness and attractiveness among girls in China, who believe that “thin is beautiful” [41]. Many girls engage in physical activities and practices, such as yoga, to maintain their figure, resulting in improved physical fitness and reduced BMI. Conversely, boys tend to maintain a normal weight rather than intentionally aiming for underweight status, as it is seen as a healthier approach. Therefore, boys with a normal weight exhibit the best physical fitness levels in the assessment.

### 4.6. Limitations

Like most studies, this study is not without limitations. The following aspects represent the limitations of this study and areas of focus for future research: (1) The data in this study are limited to Chinese adolescents aged 15–18, and no data from other countries or regions were included for comparative analysis. (2) The collection of body composition data in this study only involved height and weight measurements, which are important factors for assessing obesity. However, other significant indicators, such as body fat percentage and waist circumference [42], were not included in the data collection. These additional measurements are essential for a more comprehensive evaluation of obesity, and should be considered in future research. (3) Heart rate during physical activity is a relevant indicator of an individual’s physical fitness level [43]. For future studies, we plan to incorporate the use of smart devices to collect participants’ heart rate and other relevant data, allowing for a more accurate representation of their physical fitness levels.

## 5. Conclusions

The study examined the relationship between BMI categories and physical fitness test performances among Chinese adolescents, with a particular focus on gender differences. The findings revealed several key points:

1. Non-linear relationship: The study identified a significant non-linear relationship between BMI levels and physical fitness test scores. This suggests that there is an optimal BMI range for achieving higher physical fitness performance, while deviating from this range, either towards underweight or overweight/obesity, can negatively impact physical fitness;
2. Gender differences: Gender differences were observed in physical fitness and overall health outcomes. Normal-weight boys achieved the highest physical fitness test scores, and demonstrated better overall health levels compared to boys in other weight categories. On the other hand, underweight and normal-weight girls outperformed overweight and obese girls in physical fitness tests, with underweight girls scoring slightly higher than those with normal weight;
3. Cultural influence: The study suggests that the cultural associations between thinness and beauty among girls in China may contribute to the observed gender differences. The pressure to maintain a slim figure among girls might lead to a higher prevalence of underweight individuals, which could explain their better physical fitness performance.

## 6. Recommendations

1. BMI education and awareness: Public health interventions should prioritize raising awareness about the importance of maintaining a healthy BMI for optimal physical fitness. This could involve educating adolescents, parents, and educators about the potential risks associated with both underweight and overweight/obesity, emphasizing the need for a balanced approach to weight management;
2. Gender-specific approaches: Physical education programs should consider implementing gender-specific strategies to address the unique challenges faced by boys and girls. For boys, promoting healthy weight maintenance and providing opportunities for physical activity could further enhance their physical fitness. For girls, interventions should focus on fostering positive body image and self-esteem, moving away from the cultural pressure for extreme thinness;
3. Inclusive fitness programs: It is essential to design physical education programs that are inclusive and accessible to all students, regardless of their BMI category. Encouraging participation in a variety of physical activities, promoting teamwork, and focusing on individual progress rather than weight-related outcomes, can create a supportive environment for all adolescents;
4. Policy support: Policymakers should consider integrating BMI monitoring and fitness assessments into school health programs. This would help identify students who may require additional support and enable targeted interventions.

By implementing these recommendations, public health interventions, physical education programs, and policies can work together to improve the physical fitness and overall health outcomes of Chinese adolescents, while also addressing the unique challenges posed by gender differences and cultural influences.

## Figures and Tables

**Table 1:** Statistical table of male students.

	Mean	Std. Err.	95% Conf. Interval	
Height (cm)	174.0	0.266	173.5	174.5
Weight (kg)	63.60	0.457	62.70	64.50
BMI	20.97	0.134	20.71	21.23
50 m sprint(s)	7.888	0.0399	7.809	7.966
1000 m run(min)	4.001	0.0263	3.949	4.052
standing long jump(cm)	216.9	1.176	214.6	219.2
Sit and reach(cm)	13.26	0.285	12.70	13.82
pull-up	9.092	0.405	8.296	9.888

**Table 2:** Statistical table of female students.

	Mean	Std. Err.	95% Conf. Interval	
Height(cm)	165.6	0.235	165.2	166.1
Weight(kg)	56.27	0.358	55.57	56.97
BMI	20.48	0.111	20.26	20.70
50 m sprint(s)	8.965	0.0414	8.884	9.046
800 m run(min)	3.814	0.0207	3.773	3.854
standing long jump(cm)	174.9	0.783	173.4	176.4
Sit and reach(cm)	15.76	0.244	15.28	16.24
bent-leg sit-up	34.02	0.454	33.13	34.91

**Table 3:** Correlation between BMI and physical fitness of different genders.

	Boys					Girls				
	50 m	1000 m	s-l-j	S-a-r	P-u	50 m	800 m	s-l-j	s-l-j	P-u
overweight	0.716 ***^+++^	0.438 ^+++^	0.574 ***^+++^	0.344 ***	0.735 ***^+++^	0.279 *^+++^	0.668 *^+++^	0.131 ^+++^	0.091	0.276 *^+^
	(6.25)	(1.60)	(5.77)	(3.48)	(6.84)	(2.14)	(2.15)	(1.10)	(0.79)	(2.33)
underweight	0.530 ***^+++^	0.800 **	0.701 ***^+++^	0.251	1.089 ***	1.090 ***^+++^	1.710 ***	0.981 ***^++^	0.066	0.490 ***
	(3.58)	(2.64)	(5.49)	(1.96)	(8.44)	(7.73)	(5.08)	(7.75)	(0.54)	(3.86)
normal weight	1.052 ***	1.162 ***	1.096 ***	0.407 ***	1.028 ***	0.721 ***	1.443 ***	0.723 ***	0.230 *	0.462 ***
	(11.18)	(5.22)	(13.50)	(5.06)	(11.70)	(6.89)	(5.67)	(7.54)	(2.48)	(4.85)

Note: Obesity as reference * *p* < 0.05; ** *p* < 0.01; *** *p* < 0.001. Standard form as reference *^+^ p* < 0.05; *^++^ p* < 0.01; *^+++^ p* < 0.001 (coefficients not shown). t statistics in parentheses. 50 m = 50 m sprint; 1000 m = 1000 m run; s-l-j = standing long jump; S-a-r = sit and reach; P-u = pull-up; 800 m = 800 m run.

**Table 4:** Robustness test.

	Boys					Girls				
	50 m	1000 m	s-l-j	S-a-r	P-u	50 m	800 m	s-l-j	s-l-j	P-u
overweight	0.829 ***	0.649 *	0.566 ***	0.396 ***	0.719 ***	0.378 **	0.626	0.145	0.079	0.295 *
	(6.42)	(2.07)	(5.00)	(3.53)	(6.12)	(2.61)	(1.80)	(1.09)	(0.61)	(2.24)
underweight	0.639 ***	0.820 *	0.690 ***	0.381 *	1.107 ***	1.036 ***	1.606 ***	0.975 ***	0.051	0.568 ***
	(3.59)	(2.39)	(4.51)	(2.51)	(7.50)	(6.35)	(4.03)	(6.60)	(0.36)	(3.87)
Normal weight	1.129 ***	1.293 ***	1.100***	0.474 ***	1.000 ***	0.724 ***	1.350 ***	0.715 ***	0.256 *	0.503 ***
	(10.82)	(5.18)	(12.10)	(5.28)	(10.69)	(6.26)	(4.70)	(6.71)	(2.47)	(4.75)

Note: * p < 0.05, ** p < 0.01, *** p < 0.001, t statistics in parentheses. 50 m = 50 m sprint; 1000 m = 1000 m run; s-l-j = standing long jump; S-a-r = Sit and reach; P-u = pull-up;800 m = 800 m run.

## Data Availability

Please contact the corresponding authors to obtain the data.
